# Supplementary material for: Single molecule high-throughput footprinting of small and large DNA ligands
Source: Nat Commun. 2017 Aug 21;8:304. doi: 10.1038/s41467-017-00379-w (PMC5563512; doi:10.1038/s41467-017-00379-w)
Supplement: Supplementary file 1 — Supplementary Information [file 41467_2017_379_MOESM1_ESM.pdf]

File name: Supplementary Information
Description: Supplementary Figures, Supplementary Tables and Supplementary References.

File name: Peer Review File
Description:

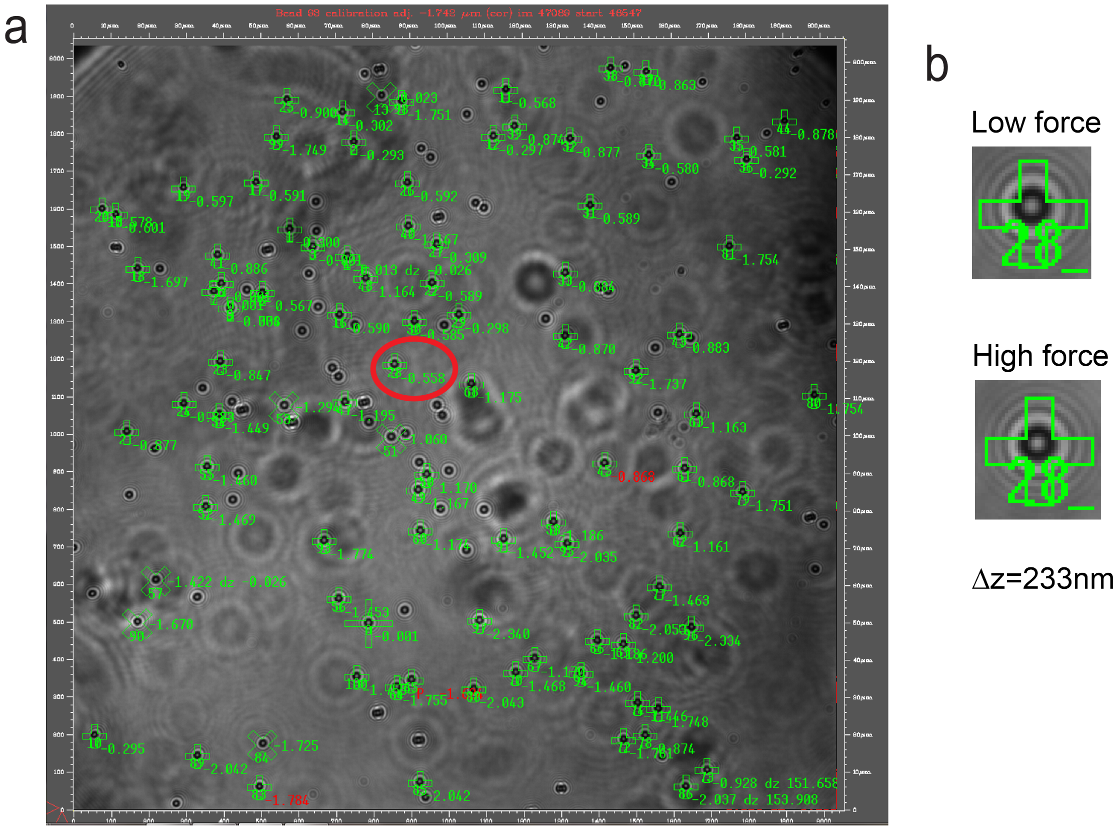

Supplementary Figure 1: Magnetic tweezers field of view. (a) Field of view of a typical experiment. (b) Details of the diffraction image of one bead, at two positions of the magnets, corresponding to a force where the hairpin is closed (low force) and a force where the hairpin is open (high force). The difference on molecular extension between the two forces, can be deduced from the analysis on the diffraction pattern with nm accuracy [1].

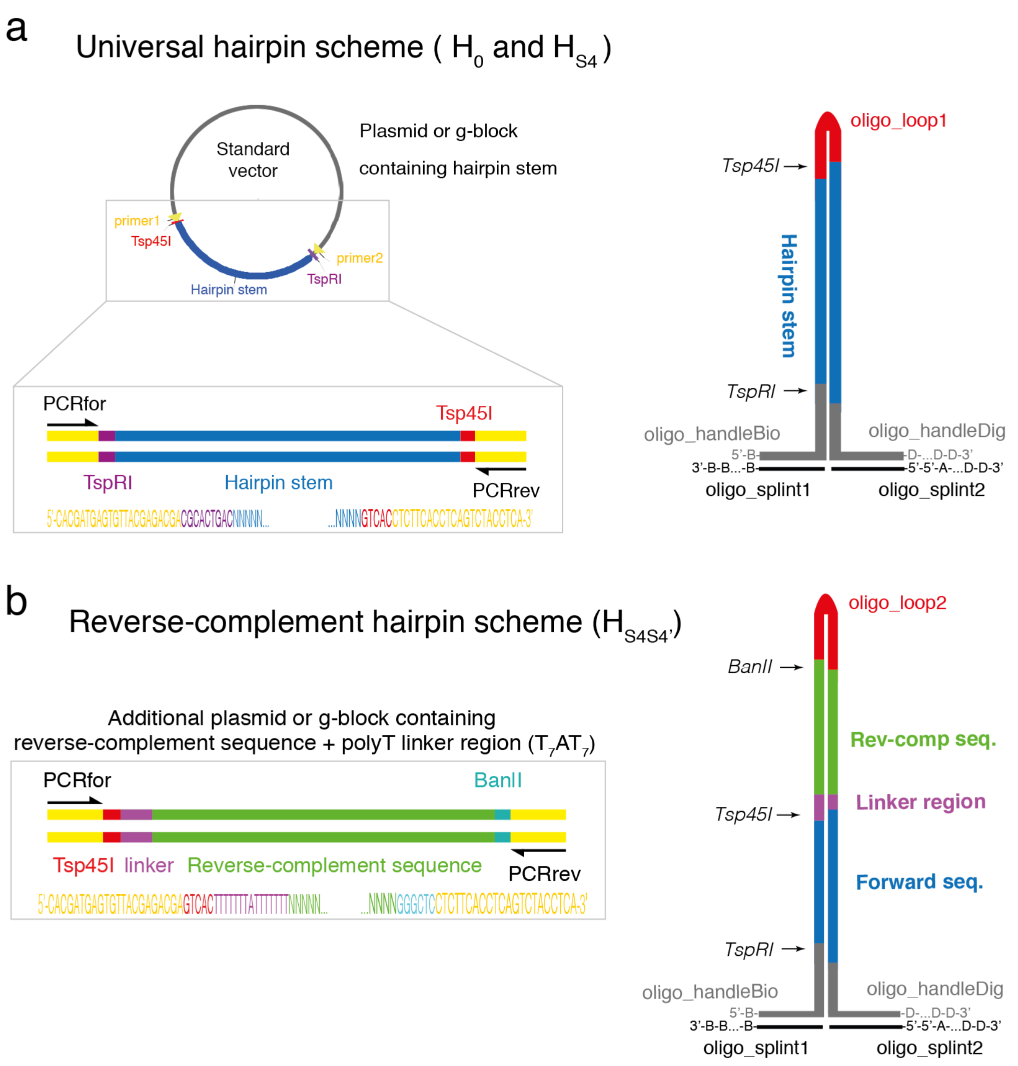

Supplementary Figure 2: Schematics of the different molecular constructs used to generate hairpins H_0, H_{s4} and $H_{s4s4'}$. (a) The sequence that will become the stem of the hairpin (blue) is embedded between the restriction sites of TspRI (purple) and Tsp45I (red) and two primer binding sites (yellow). This region of interest is purchased as a g-block or cloned into a standard commercial vector. The DNA hairpin is assembled by ligating a set of oligonucleotides (red, gray, black) to the PCR-amplified and digested fragment from the left panel. The oligonucleotides that create the handles are purchased biotinylated or are differentially end-labelled with digoxigenins and biotins using terminal transferase. The oligo splint2 uses a block 3'-end and 5'-5' inversion to be tailed at the appropiate end. (b) To create the forward + reverse-complement hairpin, an additional g-block is used. The fragment contains the reverse-complement of the original sequence embedded between a polyT linker (purple), the restriction sites of Tsp45I (red) and BanII (cyan), and two primer binding sites (yellow). The purpose of the linker is to avoid misfolding of the hairpin into a cruciform structure during pulling experiments. The fragment is PCR-amplified and digested, and the final construct is assembled and ligated in two steps. Important aspects for the synthesis are the use of non-palindromic restriction enzymes to assemble the hairpins, gel-purification after the digestion steps and final assembly of the construct (to remove excess oligos), and ensuring that the recognition sequences are not be present in the sequence of interest. The specific sequences used are depicted in Supplementary Table 1 using the same color scheme.

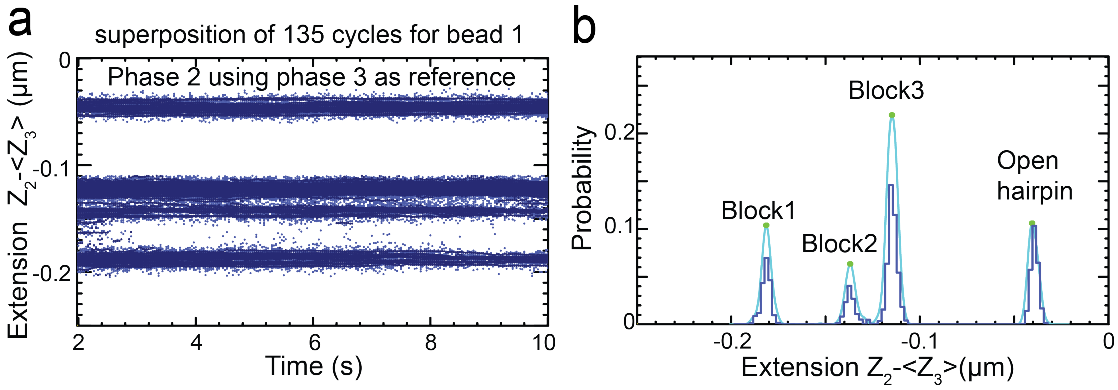

Supplementary Figure 3: Differential measurement of molecular extension. (a) Differential measurement of the molecular extension of bead 1(with respect the fixed bead) during phase two (F_{test} phase) Z_2 where we subtract the average value of the the molecular extension in phase 3 (F_{high} phase) Z_3. The results shown are the superposition of the data from 135 cycles. Subtraction of the extension in phase 3 improves the alignment between the data from different cycles. (b) Histogram of the molecular extension in panel (a) that shows different peaks. Conversion from the measured extension to base-pairs leads to the results shown in the Fig. 2b in the main text.

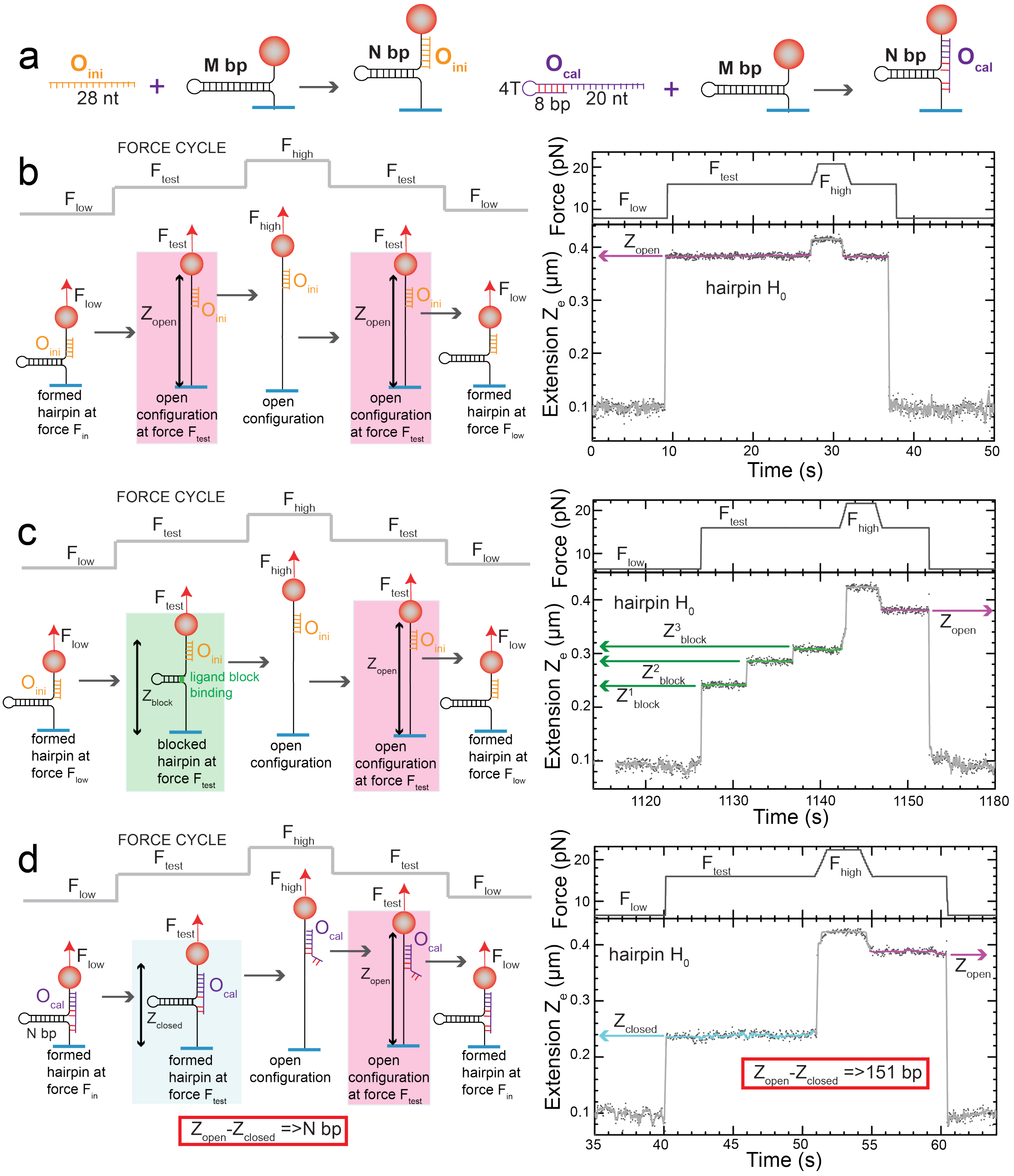
a
O ini
28 nt
M bp
N bp
O ini
4T
O cal
8 bp
20 nt
M bp
N bp
O cal
b
FORCE CYCLE
F low
F test
F high
F test
F low
formed hairpin at force F in
open configuration at force F test
Z open
open configuration
open configuration at force F test
formed hairpin at force F low
Force (pN)
Extension Z e (μm)
Z open
hairpin H 0
Time (s)
c
FORCE CYCLE
formed hairpin at force F low
ligand block binding
Z block
blocked hairpin at force F test
open configuration
open configuration at force F test
formed hairpin at force F low
hairpin H 0
Z 3 block
Z 2 block
Z 1 block
Z open
Time (s)
d
FORCE CYCLE
O cal
N bp
formed hairpin at force F in
Z closed
formed hairpin at force F test
open configuration
open configuration at force F test
formed hairpin at force F in
Z open -Z closed =>N bp
hairpin H 0
Z closed
Z open
Z open -Z closed =>151 bp
Time (s)

Supplementary Figure 4 ***(previous page)*****: Conversion from measured distances (in nanometers) to positions (in bps).** (a) Schematics of the two blocking oligonucleotides, $\mathrm{O_{ini}}$ and $\mathrm{O_{cal}}$, used in the experiments and their binding to the hairpin. The blocking oligonucleotide $\mathrm{O_{ini}}$ has 28 bases complementary to a region of the hairpin sequence. When hybridizing to the initial hairpin construct, it reduces the size of the hairpin from M to N bp. In the case of H_0 N =151bp. The blocking oligonucleotide $\mathrm{O_{cal}}$ has the same 28 bases as $\mathrm{O_{ini}}$ followed by a TTTT tetranucleotide and 8 bases complementary to the 8 last bases. Like $\mathrm{O_{ini}}$, $\mathrm{O_{cal}}$ reduces the size of the hairpin from M to N bp. (b) When using the blocking oligonucleotide $\mathrm{O_{ini}}$, that is only complementary to one strand, and applying the FC protocol, hairpin H_0 unzips in one step when the force is increased to F_{test} (left panel schematics, right panel experimental trace). $\mathrm{O_{ini}}$ is used to generate the H_0 hairpin, with 151bp, from an original longer hairpin. (c) When adding a ligand and applying the FC protocol, hairpin H_0 shows blockages during unzipping at F_{test}, generated by the DNA stabilization due to DNA/ligand interaction (left panel schematics, right panel experimental trace). In order to convert the extension of blockages Z^i_{block} to bp position we use the conversion factor I determined in the protocol shown in last panels. (d) The conversion factor I between number of open bps and molecular extension is experimentally determined by performing the FC protocol with the blocking oligonucleotide $\mathrm{O_{cal}}$, that delays the opening of the hairpin structure at F_{test} and allows measuring the extension of the formed hairpin Z_{closed} as well as the extension of the unfolded hairpin Z_{open} at the same force, F_{test}, at which the binding of ligands is tested. Left panel shows the schematics of the assay. Right panel shows an experimental trace obtained with hairpin H_0 and the blocking oligonucletide, when applying the FC protocol. For hairpin H_0, that has 151bp of length, the conversion factor is computed as $I = 151$ bp/ $(Z_{\mathrm{open}} - Z_{\mathrm{closed}})$. We also include a correction to take into account the small extension change when the blocking oligonucleotide is bound or unbound. The same strategy is used for hairpins H_{s4} and $H_{s4s4'}$.

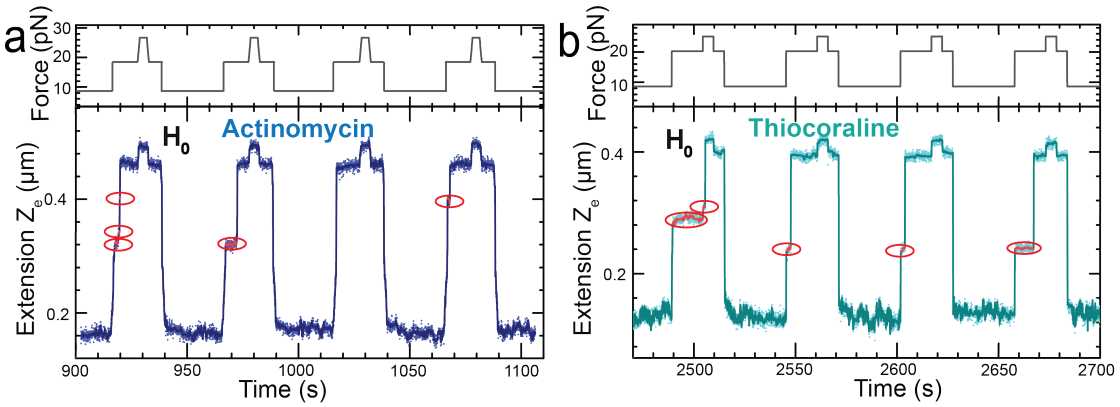

Supplementary Figure 5: Characteristic molecular extension traces for Actinomycin and Thiocoraline. Molecular extension traces for hairpin H_0 when applying a FC protocol in presence of intercalators Actinomycin (a) and Thiocoraline (b). The binding events are circled in red. The distributions of blockage positions are shown in Figure 3a in the main text

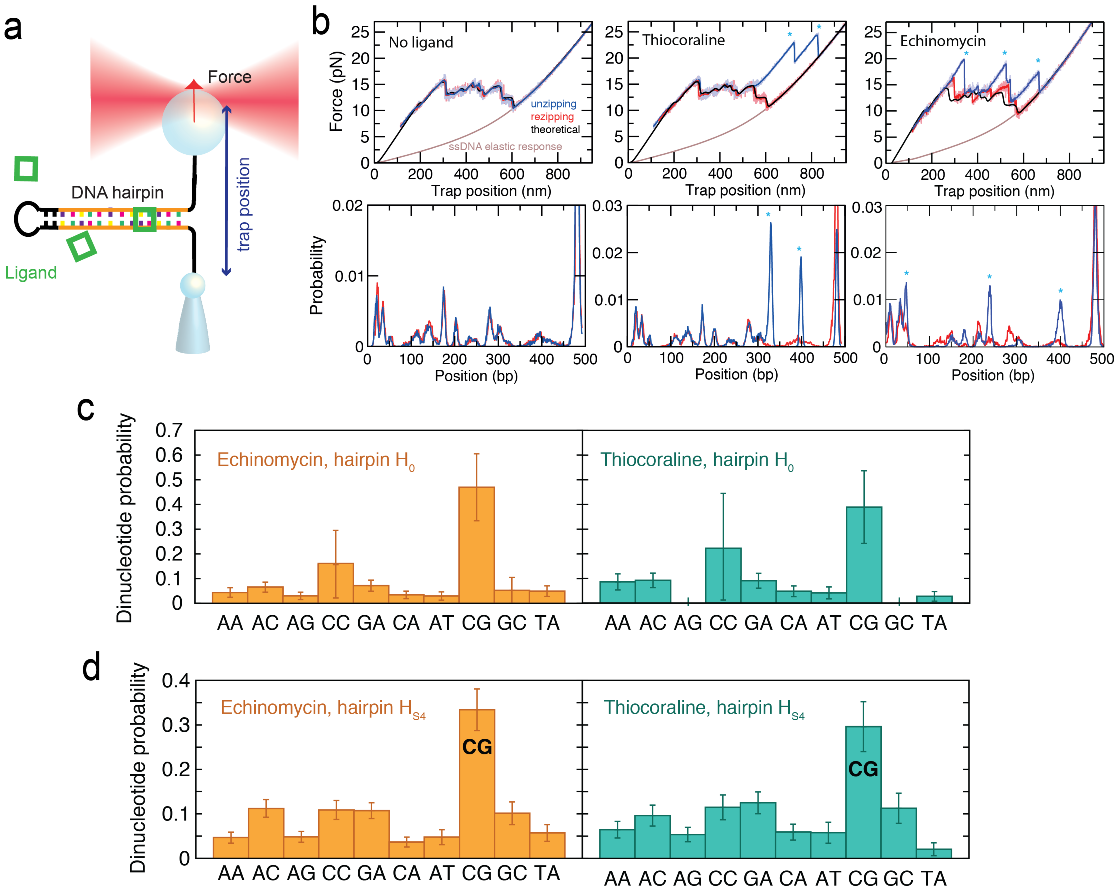

Supplementary Figure 6: Mechanical footprinting using optical tweezers. (a) Schematics of the optical tweezers experimental setup. (b) Top panel shows the force extension curve of hairpin H_0 without any ligand (left), in the presence of Thiocoraline (middle) and Echinomycin (right). Force-extension curves in the presence of both ligands show force peaks during DNA unzipping (blue curve) that correspond to binding events (indicated with cyan dots). The position (in bp) of the peaks can be determined by correlating the unzipping curve to a theoretical curve (black) as explained in [2]. Bottom panel shows the position of the binding events (indicated with a cyan asterisk) is determined by Gaussian fitting of those peaks observed in the unzipping probability plot (blue) not present in the rezipping curve (red). (c) Preferred recognition sequence of Echinomycin (left) and Thiocoraline (right) using hairpins H_0 (top) and H_{S4} (bottom) obtained from the optical tweezers experiments. The sequence specificity of each ligand is obtained by performing a correlation analysis of the binding position observed in N>60 pulling curves [2].

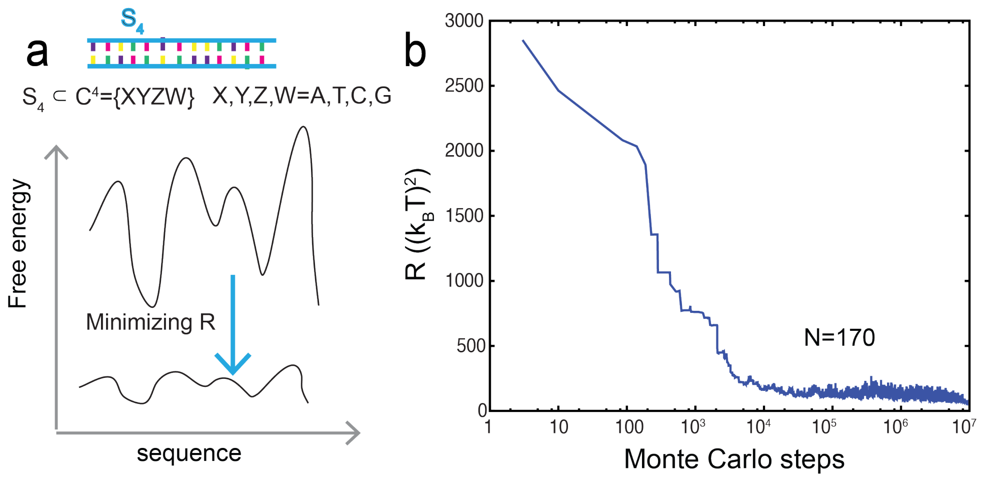

Supplementary Figure 7: Design of hairpins with a flat free-energy landscape using Monte Carlo simulations. (a) Schematics of the sequence S_4 designed for selectivity measurements of ligands that bind or recognize 4 or less bps. The sequence is generated by a Monte Carlo simulation (Methods in main text), imposing that it must include all tetranucleotide combinations at least once and not more than twice. Moreover, the free energy profile $G_f(n)$ is designed to be pretty flat by minimizing the roughness R. (b) The roughness of the free energy landscape R (defined in Methods in the main text) as a function of the Monte Carlo steps for sequences S_4 of 170 bps in length.

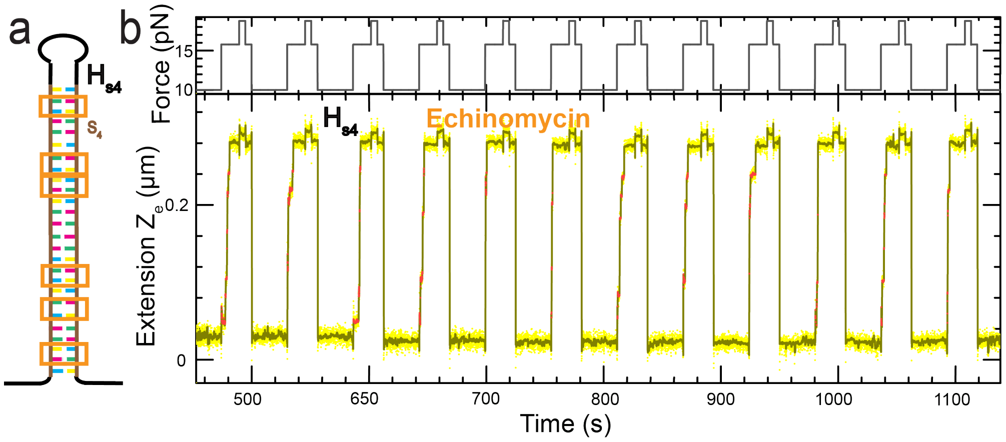

Supplementary Figure 8: Molecular extension traces for Echinomycin using hairpin H_{s4}. (a) Schematics of binding of Echinomycin to hairpin H_{s4}. (b) Molecular extension traces for hairpin H_{s4} when applying a FC protocol in presence of Echinomycin. The binding events (shown in red) are observed at many locations along the sequence. The distribution of blockage positions is shown in Figure 4c in the main text.

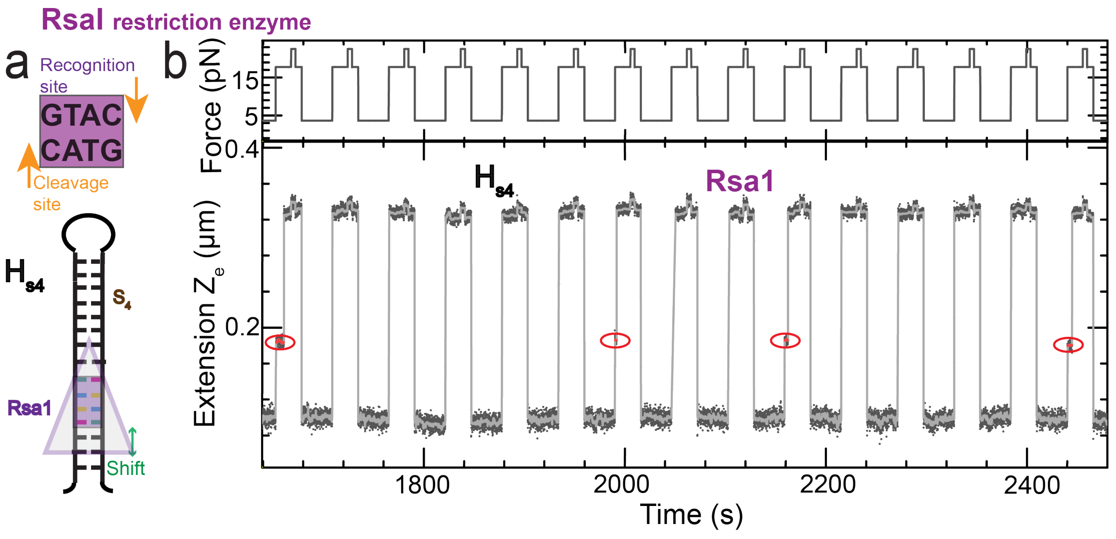

Supplementary Figure 9: Molecular extension traces for Rsa1 using hairpin H_{s4}. (a) Schematics of recognition and cleavage of the palindromic Rsa1 restriction enzyme as well as its binding to the H_{s4} hairpin. (b) Molecular extension traces for the H_{s4} hairpin, when applying a FC protocol in presence of the Rsa1 restriction enzyme. The binding events (circled in red) are observed at a single location in H_{s4} as expected.

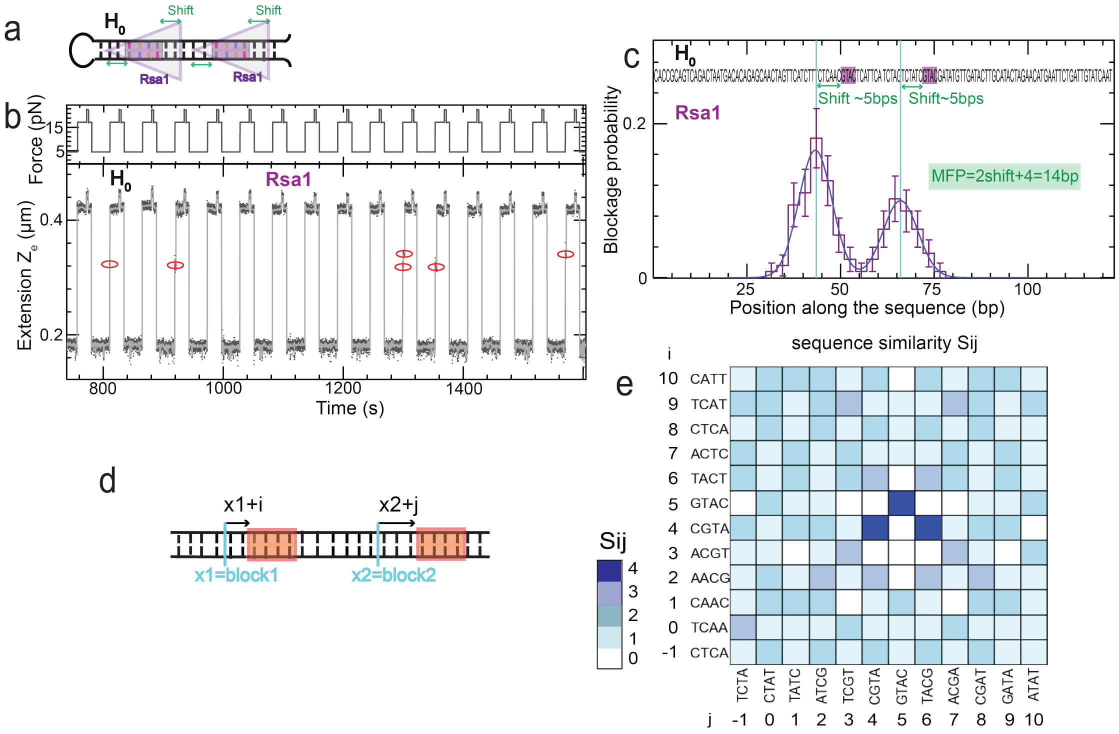

Supplementary Figure 10: Correlation analysis for Rsa1. (a) Schematics of the binding of Rsa1 restriction to hairpin H_0. (b) Molecular extension traces for hairpin H_0 when applying a FC protocol in presence of the Rsa1 restriction enzyme. The binding events (circled in red) are observed at two locations along the sequence. (c) The distribution of blockage positions presents two peaks that are fitted to Gaussian functions (Number of beads=123). The center of the Gaussian functions are shifted 5 bps from the expected enzyme restriction site. The mechanical footprint (MFP) can be estimated as twice the shift (10bps) plus the size of the recognition sequence (4bps). Error bars are inversely proportional to the square root of the number of points for each bin. (d) Schematics of the computation of the Similarity function defined in Methods in the main text, that is used to perform a sequence correlation analysis. (e) Results for the similarity function $S_{i,j}$ defined in Methods. The scale of blue denotes the degree of sequence similarity: the darkest blue color accounts for maximum similarity (e.g. all bases coincide). The results show that two repeats of GTAC or CGTA are in the vicinity of the regions where the peaks are observed, and therefore could potentially correspond to the recognition sequence of the enzyme. However, since the enzyme is palindromic we can deduce that the right recognition sequence is GTAC. The reason why the CGTA sequence appears is because both repetitions of the recognition sites along H_0 appear preceding the same base C, giving two repeated CGTA motifs. By comparing with the results obtained with other DNA substrates (such as H_{s4}) one can also infer the right recognition sequence as GTAC.

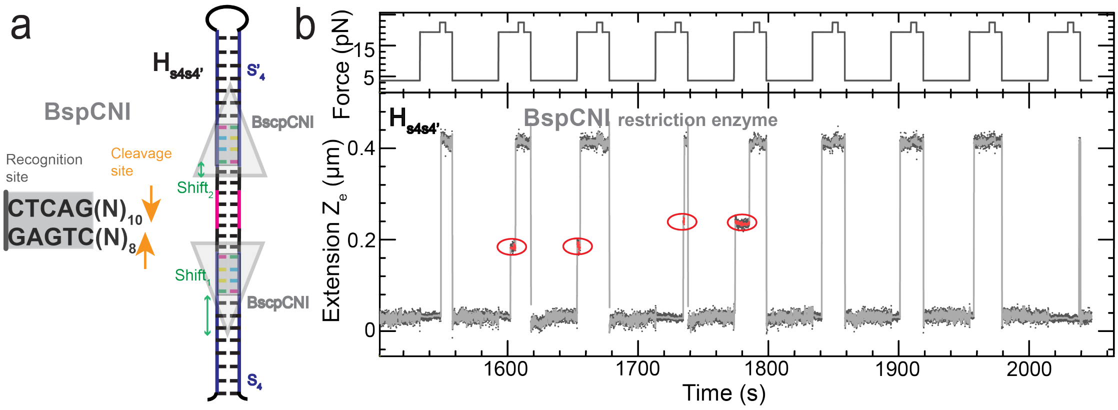

Supplementary Figure 11: Molecular extension traces for BspCNI using hairpin $H_{s4s4'}$. (a) Schematics of recognition and cleavage of BspCNI restriction enzyme as well as its binding to hairpin $H_{s4s4'}$. (b) Molecular extension traces for hairpin $H_{s4s4'}$ when applying a FC protocol in presence of the BspCNI restriction enzyme. The binding events (circled in red) are observed at two locations along the sequence. The distribution of blockage positions is shown in Figure 6a in the main text.

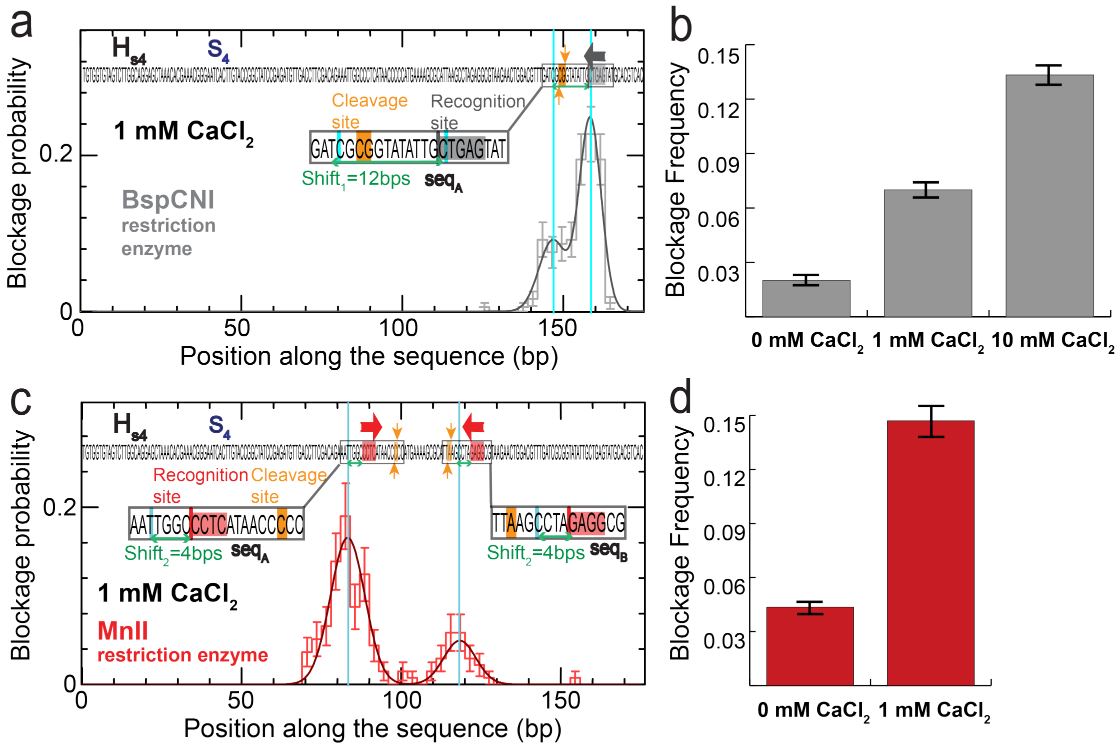

Supplementary Figure 12: Effect of divalent ion concentration on BspCNI and MnII binding. (a,c) Distribution of blockage positions obtained by applying the FC protocol to the H_{s4} hairpin in presence of the BspCNI (a, Number of beads=153) and MnII (c, Number of beads=113) restriction enzyme in presence of 1mM $CaCl_2$. The results show peaks located at the same positions measured in absence of divalent ions (Figure 6 in main text and Supplementary Table 2). Error bars are inversely proportional to the square root of the number of points for each bin. In the case of MnII, the tethered beads detach shortly after the injection of the enzyme, and indication that the Calcium ions induce cleavage. (b,d) Frequency of blockages, measured as the average number of blockages per cycle, for BspCNI (b) and MnII (d) at different $CaCl_2$ concentrations. Error bars correspond to the s.e.m.

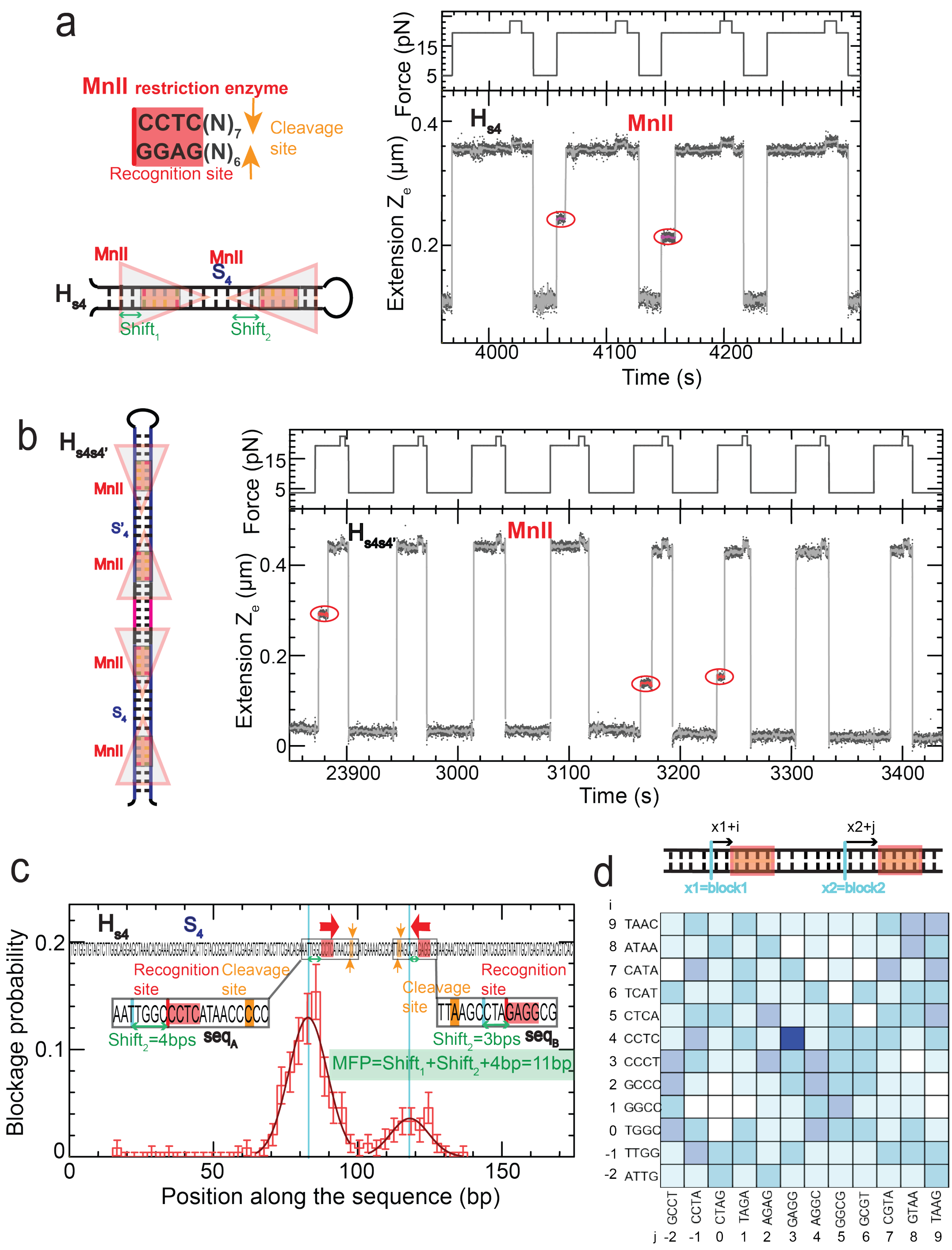
a
MnlI restriction enzyme
CCTC(N)7
GGAG(N)6
Cleavage site
Recognition site
MnlI
MnlI
S4
Hs4
Shift1
Shift2
Force (pN)
15
5
Hs4
MnlI
Extension Ze (μm)
0.4
0.2
4000
4100
4200
Time (s)
b
Hs4s4'
MnlI
S'4
MnlI
MnlI
S4
MnlI
Force (pN)
Hs4s4'
MnlI
Extension Ze (μm)
23900
3000
3100
3200
3300
3400
Time (s)
c
Hs4
S4
Recognition site
Cleavage site
Cleavage site
Recognition site
AATTGGCCCTCATAACCCCC
seqA
Shift1=4bps
TTAAGCCTAGAGGCG
seqB
Shift2=3bps
MFP=Shift1+Shift2+4bp=11bp
Blockage probability
Position along the sequence (bp)
d
x1+i
x2+j
x1=block1
x2=block2
i
9 TAAC
8 ATAA
7 CATA
6 TCAT
5 CTCA
4 CCTC
3 CCCT
2 GCCC
1 GGCC
0 TGGC
-1 TTGG
-2 ATTG
GCCT
CCTA
CTAG
TAGA
AGAG
GAGG
AGGC
GGCG
GCGT
CGTA
GTAA
TAAG
j -2 -1 0 1 2 3 4 5 6 7 8 9

Supplementary Figure 13 ***(previous page)*****: Correlation analysis for MnII.** (a) Schematics of recognition and cleavage of MnII restriction enzyme as well as its binding to hairpin H_{s4} (left) and molecular extension traces for hairpin H_{s4} when applying a FC protocol in presence of the MnII restriction enzyme (right). The binding events (circled in red) are observed at two locations along the sequence. Schematics of the binding of MnII to hairpin $H_{s4s4'}$ (left) and molecular extension traces for hairpin $H_{s4s4'}$ when applying a FC protocol in presence of the MnII restriction enzyme (right). The binding events (circled in red) are observed at different locations along the sequence. The distribution of blockage positions is shown in the Figure 6b in the main text. (c) Distribution of blockage positions obtained by applying the FC protocol to the H_{s4} hairpin in presence of the MnII restriction enzyme (Number of beads=168). Error bars are inversely proportional to the square root of the number of points for each bin. The results show two peaks that can be related to the two recognition sites present in the sequence. Since the two sites are differently oriented, the shifts associated to the enzyme head and back, shift_1 and shift_2, can be measured and so the MFP. (d) Results for the sequence correlation analysis, where the scale of blue denotes the degree of sequence similarity, $S_{i,j}$ defined in Methods: the darkest blue color designs maximum similarity. A single sequence, CCTC, presents a maximum similarity. In other words, CCTC is the only sequence that is present in the vicinity of the two regions where the peaks are observed, and can then be identified as the recognition sequence.

Name	Oligonucleotide sequence
PCRfor	5'-CAC GAT GAG TGT TAC GAG ACG A-3'
PCRrev	5'-TGA GGT AGA CTG AGG TGA AGA G-3'
oligo_loop1	5'-Pho-GTC ACT TAG TAA CTA ACA TGA TAG TTA CTT TTG TAA CTA TCA TGT TAG TTA CTA A-3'
oligo_loop2	5'-Pho-TTA GTA ACT AAC ATG ATA GTT ACT TTT GTA ACT ATC ATG TTA GTT ACT AAA GCC-3'
oligo_handleDig	5'-Pho-AAG ATC TAT TAT ATA TGT GTC TCT ATT AGT TAG TGG TGG AAA CAC AGT GCC AGC GC-3'
oligo_handleBio	5'-Bio-GAC TTC ACT AAT ACG ACT CAC TAT AGG GAA ATA GAG ACA CAT ATA TAA TAG ATC TTC GCA CTG AC-3'
oligo_splint1	5'-TCC CTA TAG TGA GTC GTA TTA GTG AAG TC-3'
oligo_splint2	3'-AAA AA-5'-5'-GCG CTG GCA CTG TGT TTC CAC CAC TAA C(SpC3)-3'
Embedding g-block forward	5'-CACGATGAGTGTTACGAGACGA CGCACTGACNNN.....NNN TTTTTTTATTTTTTTNNN.....NNNGGGCTCCTCTTCACCTCAGTCTACCTCA-3'
Embedding g-block rev-comp	5'-CACGATGAGTGTTACGAGACGAGTCAC TTTTTTTATTTTTTTNNN.....NNNGGGCTCCTCTTCACCTCAGTCTACCTCA-3'

Supplementary Table 1: Oligonucleotides used for hairpin synthesis. Oligonucleotides used for the DNA hairpin synthesis protocols depicted in Supplementary Figure 1 (color of embedding blocks follows the notation used in the figure).

Figure	N beads	N points	peak	Gaussian fit
2b	1	39.088	peak 1	$x_1^0 = 2$ bp, $\sigma_1 = 2$ bp, $w_1/w_1 = 1$
2b	1	39.088	peak 2	$x_2^0 = 46$ bp, $\sigma_2 = 2$ bp, $w_2/w_1 = 0.6$
2b	1	39.088	peak 3	$x_3^0 = 71$ bp, $\sigma_3 = 2$ bp, $w_3/w_1 = 2.2$
2b	1	39.088	peak 4	$x_4^0 = 151$ bp, $\sigma_4 = 2$ bp, $w_4/w_1 = 1.4$
2g	1	6907	peak 1	$x_1^0 = 301$ nm, $\sigma_1 = 2$ nm, $w_1/w_1 = 1$
2g	1	6907	peak 2	$x_2^0 = 337$ nm, $\sigma_2 = 4$ nm, $w_2/w_1 = 0.5$
2g	1	6907	peak 3	$x_3^0 = 356$ nm, $\sigma_3 = 2$ nm, $w_3/w_1 = 1.3$
3a upper panel	93	162	peak 1	$x_1^0 = 1$ bp, $\sigma_1 = 2$ bp, $w_1/w_1 = 1$
3a upper panel	93	162	peak 2	$x_2^0 = 46$ bp, $\sigma_2 = 2$ bp, $w_2/w_1 = 1.3$
3a upper panel	93	162	peak 3	$x_3^0 = 71$ bp, $\sigma_3 = 4$ bp, $w_3/w_1 = 1.6$
3a middle panel	174	266	peak 1	$x_1^0 = 4$ bp, $\sigma_1 = 2$ bp, $w_1/w_1 = 1$
3a middle panel	174	266	peak 2	$x_2^0 = 24$ bp, $\sigma_2 = 2$ bp, $w_2/w_1 = 0.5$
3a middle panel	174	266	peak 3	$x_3^0 = 88$ bp, $\sigma_3 = 3$ bp, $w_3/w_1 = 1$
3a lower panel	127	205	peak 1	$x_1^0 = 2$ bp, $\sigma_1 = 3$ bp, $w_1/w_1 = 1$
3a lower panel	127	205	peak 2	$x_2^0 = 49$ bp, $\sigma_2 = 4$ bp, $w_2/w_1 = 1.4$
3a lower panel	127	205	peak 3	$x_3^0 = 72$ bp, $\sigma_3 = 4$ bp, $w_3/w_1 = 1.5$
4c	182	783	peak 1	$x_1^0 = 28$ bp, $\sigma_1 = 2$ bp, $w_1/w_1 = 1$
4c	182	783	peak 2	$x_2^0 = 48$ bp, $\sigma_2 = 3$ bp, $w_2/w_1 = 1.3$
4c	182	783	peak 3	$x_3^0 = 69$ bp, $\sigma_3 = 4$ bp, $w_3/w_1 = 0.8$
4c	182	783	peak 4	$x_4^0 = 69$ bp, $\sigma_4 = 4$ bp, $w_4/w_1 = 0.7$
4c	182	783	peak 5	$x_5^0 = 107$ bp, $\sigma_5 = 2$ bp, $w_5/w_1 = 0.2$
4c	182	783	peak 6	$x_6^0 = 124$ bp, $\sigma_6 = 4$ bp, $w_6/w_1 = 1.5$
4c	182	783	peak 7	$x_7^0 = 137$ bp, $\sigma_6 = 2$ bp, $w_7/w_1 = 0.9$
4c	182	783	peak 8	$x_8^0 = 147$ bp, $\sigma_8 = 2$ bp, $w_8/w_1 = 0.6$
4c	182	783	peak 9	$x_9^0 = 168$ bp, $\sigma_9 = 2$ bp, $w_9/w_1 = 0.9$
5c	72	72	peak 1	$x_1^0 = 41$ bp, $\sigma_1 = 4$ bp, $w_1/w_1 = 1$
6a	155	194	peak 1	$x_1^0 = 146$ bp, $\sigma_1 = 7$ bp, $w_1/w_1 = 1$
6a	155	194	peak 2	$x_2^0 = 161$ bp, $\sigma_2 = 6$ bp, $w_2/w_1 = 1.2$
6a	155	194	peak 3	$x_3^0 = 194$ bp, $\sigma_3 = 5$ bp, $w_3/w_1 = 1.4$
6a	155	194	peak 4	$x_4^0 = 209$ bp, $\sigma_4 = 4$ bp, $w_4/w_1 = 0.6$
6b	139	243	peak 1	$x_1^0 = 83$ bp, $\sigma_1 = 9$ bp, $w_1/w_1 = 1$
6b	139	243	peak 2	$x_2^0 = 119$ bp, $\sigma_2 = 7$ bp, $w_2/w_1 = 0.2$
6b	139	243	peak 3	$x_3^0 = 232$ bp, $\sigma_3 = 9$ bp, $w_3/w_1 = 0.6$
6b	139	243	peak 4	$x_4^0 = 267$ bp, $\sigma_4 = 8$ bp, $w_4/w_1 = 0.2$
S10c	123	158	peak 1	$x_1^0 = 43$ bp, $\sigma_1 = 4$ bp, $w_1/w_1 = 1$
S10c	123	158	peak 2	$x_2^0 = 65$ bp, $\sigma_2 = 5$ bp, $w_2/w_1 = 0.7$
S12a	153	185	peak 1	$x_1^0 = 146$ bp, $\sigma_1 = 4$ bp, $w_1/w_1 = 1$
S12a	153	185	peak 2	$x_2^0 = 159$ bp, $\sigma_2 = 4$ bp, $w_2/w_1 = 2$
S12c	112	137	peak 1	$x_1^0 = 83$ bp, $\sigma_1 = 5$ bp, $w_1/w_1 = 1$
S12c	112	137	peak 2	$x_2^0 = 118$ bp, $\sigma_2 = 4$ bp, $w_2/w_1 = 0.3$
S13c	168	241	peak 1	$x_1^0 = 83$ bp, $\sigma_1 = 7$ bp, $w_1/w_1 = 1$
S13c	168	241	peak 2	$x_2^0 = 118$ bp, $\sigma_2 = 7$ bp, $w_2/w_1 = 0.3$

Supplementary Table 2: Summary of Gaussian fits. Statistics and parameters of the Gaussian fits presented in the different Figures in the main text and Supplementary Materials. Each peak i is fitted with a Gaussian function $g_i(x) = w_i \exp[(x - x_i^0)^2/(2\sigma_i^2)]$.

Molecule	F_{hop} (pN)	i, j	ln[$p_{\rm i}/p_{\rm j}$]	$\Delta\Delta G^0_{\rm i,j}(k_{\rm B}{\rm T})$	$\Delta G^{ssDNA}_{\rm i,j}(F)(k_{\rm B}{\rm T})$	$\Delta G_{\rm int}(k_{\rm B}{\rm T})$
1	15.3	1, 2	1.03	104.4	110.4	
1	15.3	1, 3	0.54	149.5	163.3	7.1
2	15	1, 2	2.29	104.4	107.9	
2	15	1, 3	9.63	149.5	158.55	7.2
3	15.36	1, 2	0.09	104.4	111.1	
3	15.36	1, 3	-1.17	149.5	164.2	6.8
4	15.43	1, 2	0.96	104.4	112.	
4	15.43	1, 3	-0.5	149.5	164.8	7.9
5	15.7	1, 2	-2.1	104.4	114.7	
5	15.7	1, 3	-6.3	149.5	169.6	7.5
Average						**7.3**

Supplementary Table 3: Free energy contributions to the different partially unzipped H_0 DNA configurations in presence of Echinomycin. The ratio of probabilities $p_{\rm i}/p_{\rm j}$ is computed as the ratio between the weights of the Gaussian fits, A_i/A_j, in the histogram of molecular extension at F_{hop} (main text Fig. 2g). $\Delta\Delta G^0_{\rm i,j} = \Delta G^0_{\rm i} - \Delta G^0_{\rm j}$ is the free energy at zero force associated to the stretch of $n_i - n_j$ bps (with n_i and n_j being the number of bps formed in states i and j) and is estimated using the nearest neighbourg free energies from Mfold [3]. $\Delta G^{\rm ssDNA}_{\rm i,j}(f)$ is the stretching free energy associated to the $n_i - n_j$ unzipped bps. It can be written as $\Delta G^{ssDNA}_{\rm i,j}(F) = (n_i - n_j)G^{\rm ssDNA}(F)$, where $G^{\rm ssDNA}(F)$ is the free energy associated to the stretching of two nucleotides of ssDNA released upon unzipping a single bp at force F, $G^{\rm ssDNA}(F) = \int x(F)dF$. This latter contribution can be computed using the FJC model with paramaters given in [4, 5]. $\Delta G_{\rm int}$ is the Echinomycin binding energy estimated as: $\Delta G_{\rm int} = k_B T ln[p_j/p_i] - \Delta\Delta G^0_{\rm i,j} + (n_i - n_j)G^{\rm ssDNA}(F)$. Since the applied force $F = F_{hop}$ is slightly different for each bead-DNA tether and it is not directly measured in the experiment, for each molecule we estimate F_{hop}, as the force that verifies that $\Delta G_{\rm int}$ obtained for $(i, j) = 1, 2$ equals to that obtained for $(i, j) = 1, 3$. In this way, for each molecule where hopping is observed we get a single estimate of $\Delta G_{\rm int}$.

Supplementary References

[1] Gosse, C. & Croquette, V. Magnetic tweezers: micromanipulation and force measurement at the molecular level. *Biophys. J.* **82**, 3314–3329 (2002).

[2] Camunas-Soler, J. *et al.* Single-molecule kinetics and footprinting of DNA bis-intercalation: the paradigmatic case of thiocoraline. *Nucleic Acids Res.* **43**, 2767–2779 (2015).

[3] SantaLucia, J. A unified view of polymer, dumbbell, and oligonucleotide DNA nearest-neighbor thermodynamics. *Proc. Natl. Acad. Sci. USA* **95**, 1460–1465 (1998).

[4] Huguet, J. M. *et al.* Single-molecule derivation of salt dependent base-pair free energies in DNA. *Proc. Natl. Acad. Sci. USA* **107**, 15431–15436 (2010).

[5] Bosco, A., Camunas-Soler, J. & Ritort, F. Elastic properties and secondary structure formation of single-stranded DNA at monovalent and divalent salt conditions. *Nucleic Acids Res.* **42**, 2064 (2014).
